# The Role of Non-Coding RNAs in Autophagy During Carcinogenesis

**DOI:** 10.3389/fcell.2022.799392

**Published:** 2022-03-02

**Authors:** Patricia de la Cruz-Ojeda, Rocío Flores-Campos, Elena Navarro-Villarán, Jordi Muntané

**Affiliations:** ^1^ Institute of Biomedicine of Seville (IBiS), Hospital University “Virgen del Rocío”/CSIC/University of Seville, Seville, Spain; ^2^ Department of Medical Physiology and Biophysics, University of Seville, Seville, Spain; ^3^ Networked Biomedical Research Center Hepatic and Digestive Diseases (CIBEREHD o Ciberehd), Institute of Health Carlos III, Madrid, Spain

**Keywords:** autophagy, beclin-1, cancer, miRNA, lncRNA

## Abstract

Macroautophagy (autophagy herein) is a cellular stress response and a survival pathway involved in self-renewal and quality control processes to maintain cellular homeostasis. The alteration of autophagy has been implicated in numerous diseases such as cancer where it plays a dual role. Autophagy serves as a tumor suppressor in the early phases of cancer formation with the restoration of homeostasis and eliminating cellular altered constituents, yet in later phases, autophagy may support and/or facilitate tumor growth, metastasis and may contribute to treatment resistance. Key components of autophagy interact with either pro- and anti-apoptotic factors regulating the proximity of tumor cells to apoptotic cliff promoting cell survival. Autophagy is regulated by key cell signaling pathways such as Akt (protein kinase B, PKB), mammalian target of rapamycin (mTOR) and AMP-activated protein kinase (AMPK) involved in cell survival and metabolism. The expression of critical members of upstream cell signaling, as well as those directly involved in the autophagic and apoptotic machineries are regulated by microRNAs (miRNAs) and long non-coding RNAs (lncRNAs). Consequently, non-coding RNAs play a relevant role in carcinogenesis and treatment response in cancer. The review is an update of the current knowledge in the regulation by miRNA and lncRNA of the autophagic components and their functional impact to provide an integrated and comprehensive regulatory network of autophagy in cancer.

## Introduction

Autophagy is a catabolic degradation process which consists in the recycling or degradation of cellular components through membrane-trafficking pathways. Autophagy is usually divided into three categories: macroautophagy, microautophagy or chaperone-mediated autophagy (CMA) (Shen et al., 2015). In this review, we will refer to macroautophagy as autophagy, unless clearly specified. Although it was initially thought that autophagy was a non-selective bulk degradation pathway, it is now widely accepted that there are two categories of autophagic degradation, selective or nonselective. Whereas non-selective autophagy involves the breaking down of cytoplasmic components in order to provide cells with nutrients for survival purposes, selective mechanisms target misfolded protein aggregates, damaged and excess organelles such as mitochondria, endoplasmic reticulum (ER), lipid droplets or invading bacteria and viruses (Allaire et al., 2019; de la Ballina et al., 2020; Schulze et al., 2020).

Autophagy shows both roles in health and disease. The implications of autophagy during development have been extensively studied in model organisms, which have provided huge mechanistic knowledge (Mizushima and Levine, 2010). It has been shown to be indispensable for metamorphosis (Juhász et al., 2003) and neuromuscular development (Shen and Ganetzky, 2009) in *Drosophila melanogaster*, or to promote spore formation in yeast (Clancey et al., 1993). Furthermore, autophagy supports the elimination of maternal proteins and organelles during mammalian embryonic development, allowing genetic reprogramming. For that, the induction of autophagy in mouse cloned embryos enhances viability (Shen et al., 2015).

It is widely assumed that autophagy might be induced by several stimuli, including hypoxia, energy depletion, nutrient starvation, misfolded protein aggregates, or damaged and excess organelles (Hurley and Young, 2017; Dikic and Elazar, 2018; Onorati et al., 2018). On the other hand, basal autophagy contributes to the maintenance of cell growth and homeostasis in the absence of triggering signals, even under nutrient-rich conditions. Defects in basal autophagy could lead to pathological situations such as the accumulation of misfolded proteins in Parkinson disease (Zhang et al., 2012). In cancer, autophagy shows again a dual role, being potentially pro- or anti-tumoral during carcinogenesis and treatment response. This aspect will be next assessed from a broad perspective and also related to different autophagy associated processes such as metabolic adaptations of cancer cells or ER stress.

Non-coding RNAs, such as microRNAs (miRNAs) and long non-coding RNA (lncRNA), regulate a plethora of crucial cellular events, including cell cycle, apoptosis, cell differentiation, etc. The expression of key components of upstream Akt (protein kinase B, PKB), mammalian target of rapamycin (mTOR) and AMP-activated protein kinase (AMPK) signaling, as well as those directly involved in the autophagic and apoptotic machineries are regulated by miRNAs under physiological and pathologic conditions. Moreover, dysregulation of autophagy-related miRNAs might be associated with several diseases, including cancer, neurodegeneration, cardiovascular disease and infections (Gozuacik et al., 2017). The review will identify the miRNA and lncRNA regulating the integrated signaling networking leading to autophagy in cancer.

## Molecular Basis of Autophagy

Macroautophagy is the only autophagic process that requires the formation of double membrane vesicles called autophagosomes, which engulf cellular content to be degraded after fusion with lysosomes (Mulcahy Levy and Thorburn, 2020). On the other hand, microautophagy targets for degradation cytoplasmic cargo by means of autophagic tubes (Li et al., 2012). CMA selects cytosolic proteins for translocation and degradation in lysosomes (Kaushik and Cuervo, 2018).

**FIGURE 1:**
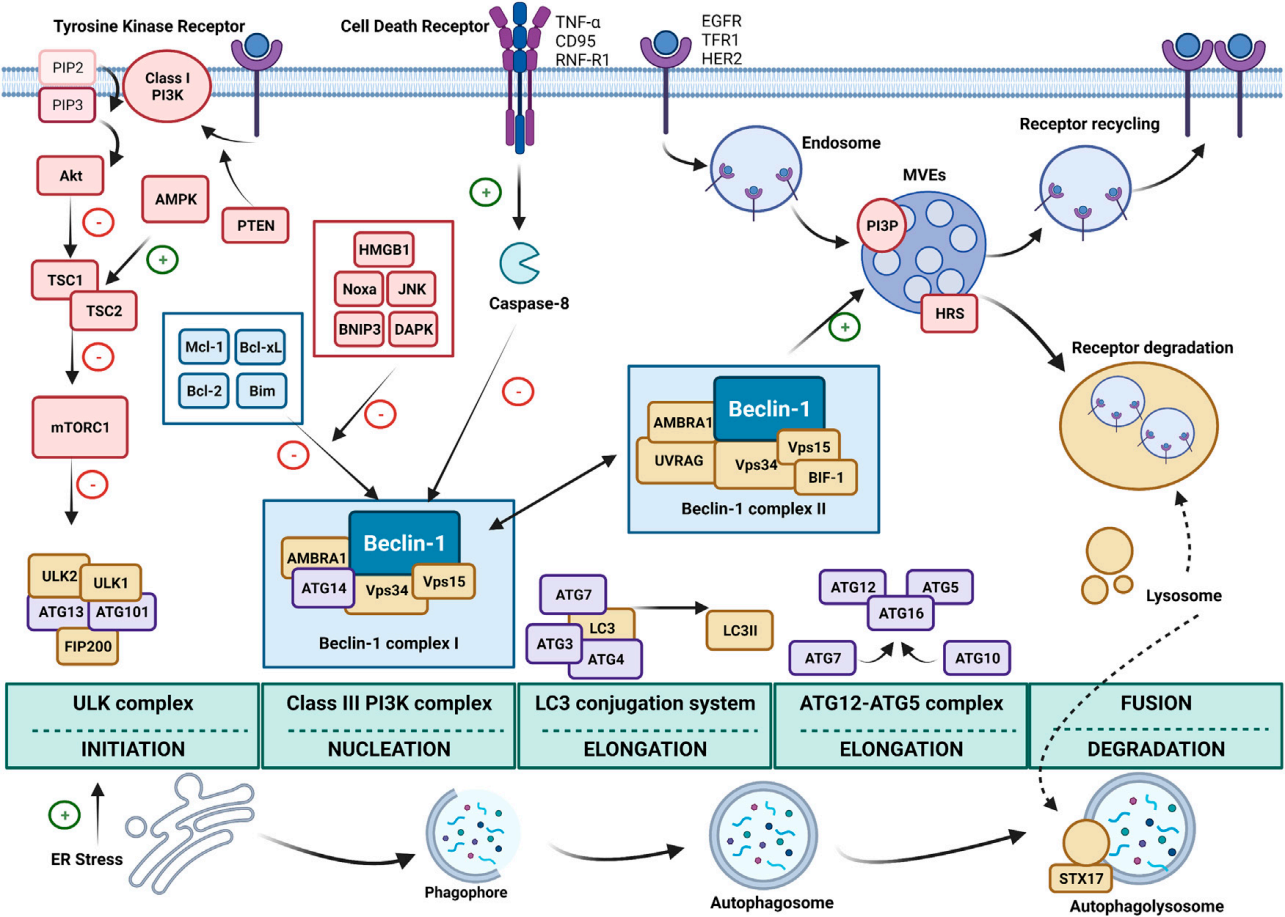
The central role of Beclin-1 during autophagy. Autophagy phases could be divided into: initiation, nucleation and formation of the phagophore, elongation and formation of the autophagosome, and fusion with the lysosome to form the autophagolysosome for degradation. The initiation step is mainly controlled by the Unc-51 like autophagy activating kinase (ULK) complex. Endoplasmic reticulum (ER) stress promotes initiation of autophagy activating the ULK complex. Also, mammalian target of rapamycin complex 1 (mTORC1) inhibits autophagy by regulating ULK1 phosphorylation. mTORC1 activity is inhibited by the Tuberous sclerosis complex 1 and 2 (TSC1, TSC2). AMP-activated protein kinase (AMPK) positively regulates autophagy by promoting TSC complex. Furthermore, tyrosine kinase receptors constitute upstream regulators of mTORC1 by promoting Akt activity, which inhibit TSC, by means of phosphatidylinositol (3,4,5)-trisphosphate (PIP3) production by phosphatidylinositol-3 kinase (PI3K) class I. The tumor suppressor phosphatase and tensin homolog (PTEN) promotes this signaling. Once the autophagic induction has been triggered, the class III PI3K complex allows the formation of the phagophore. The Beclin-1 complex is constituted by Beclin-1, ATG14, vacuolar protein sorting 34 (Vps34), vacuolar protein sorting 15 (Vps15) and autophagy protein 1 (AMBRA1). This complex I specifically focuses Beclin-1 on autophagic functions. Cell death induction by cell death receptor activation leads to caspase-8 cleavage, which ultimately inactivates Beclin-1 so that cells can undergo apoptosis. Beclin-1 interactions with Mcl-1, Bcl-2, Bcl-xL and Bim prevent its association with the pre-autophagosomal membrane, blocking autophagy. This inhibition could be reversed by high mobility group box 1 (HMGB1), c-Jun N-terminal protein kinase 1 (JNK), Noxa, BCL2/adenovirus E1B 19 kDa protein-interacting protein 3 (BNIP3) and death-associated protein kinase (DAPK) related signaling. Beclin-1 also displays non-autophagic properties when assembled into the Beclin-1 complex II, in conjunction with UV radiation resistance associated (UVRAG), BIF-1, AMBRA1, Vps34 and Vps15. In this complex, Beclin-1 participates in the endosomal pathway of epidermal growth factor receptor (EGFR), transferrin receptor type 1 (TFR1) and HER2. Receptors could be internalized into endosomes and sorted to multivesicular endosomes (MVEs). Together with phosphatidylinositol 3-phosphate (PI3P) production and recruitment of hepatocyte growth factor tyrosine kinase substrate (HRS). Beclin-1 promotes degradation of receptors after fusion with lysosomes. Otherwise, in the absence of Beclin-1, receptors would be recycled to the plasma membrane to promote survival. Beclin-1 pivots between both complexes to promote anti-tumoral functions. Other two complexes participate in the elongation of the pre-autophagosomal membrane: the LC3 conjugation system and the ATG12-ATG5 system. After formation, the autophagosome fuses with lysosomes, where syntaxin 17 (STX17) plays a major role.

Autophagy is characterized by different stages: initiation (induction and phagophore nucleation), elongation (phagophore expansion), fusion (autolysosome formation) and sealing of the autolysosome for degradation (Figure 1). Autophagy initiation begins with the activation of the complex, including the central kinase protein Unc-51-like kinase 1 (ULK1) and non-catalytic subunits ATG13 (autophagy-related protein 13), inducible coiled-coil protein 1 (FIP200) and ATG101. Autophagy induction is controlled by phosphorylation and ubiquitination of ULK1 kinase protein by mTOR complex 1 (mTORC1) and AMPK. Under nutrient-rich conditions, mTORC1 binds ULK1 and phosphorylates in Ser^757^, avoiding autophagy initiation. Phosphorylated ULK1 reduces lysosome-autophagosome fusion due to affinity loss of ULK1 for syntaxin 17 (STX17). Also, phosphorylated ULK1 enhances the interaction with heat shock cognate 71 kDa protein (HSC70) and increases CMA degradation (Hurley and Young, 2017; Cao et al., 2021).

Upon autophagy activation, ULK1 complex phosphorylates and activates the type-III phosphoinositol-3 kinase Vps34 (vacuolar protein sorting 34) that controls the nucleation stage of autophagy. The complex constituted by Vps34, Beclin-1, ATG14, vacuolar protein sorting 15 (Vps15) and activating molecule in Beclin-1-regulated autophagy protein 1 (AMBRA1) leads to the phosphorylation of membrane-associated phosphoinositol lipids (PI), resulting in the accumulation of phosphoinositol-3-phosphate (PI3P) on the cytoplasmic surface of intracellular membranes generating the named omegasome which can be originated from ER, Golgi Apparatus and the plasma membrane (Hamasaki et al., 2013; Lamb et al., 2013; Nascimbeni et al., 2017). PI3P on the membranes recruits lipid-binding proteins that mark the nucleation sites, such as phosphatidylinositol (3,4,5)-triphosphate (PIP3), double FYVE-containing protein 1 (DFCP1) and WD-repeat protein interacting with phosphoInositides (WIPI) proteins.

Two ubiquitylation-like conjugation systems are involved in the elongation of isolation membranes: ATG12-ATG5-ATG16 system and ATG8/microtubule associated protein light chain 3 (LC3)-lipid conjugation system. One of the constituents of the omegasome, WIPI2, is recruited into the complex formed by ATG12-ATG5-ATG16 due to its direct binding capacity to ATG12. ATG12-ATG5-ATG16 complex serves as an E3-like function in the second conjugation system (Kuma et al., 2002; Fujita et al., 2008). Coupling involving E1-like enzyme ATG7 and E2-like enzyme ATG3 result in the activation of ATG8 proteins such as LC3, Golgi-associated ATPase Enhancer of 16 kDa (GATE-16) and γ-aminobutyric acid receptor-associated proteins 1/2 (GABARAP1/2). ATG12-ATG5-ATG16 complex serves as an E3 ligase and facilitates conjugation of ATG8 proteins to lipid molecules such as membrane-resident phosphatidylethanolamine (PE), hence forming the membrane-bound, lipidated forms. Apart from their binding capacity with proteins with LC3 interaction region (LIR), ATG8s proteins are also required for phagophore membrane elongation and closure (Dikic and Elazar, 2018; Cao et al., 2021).

In addition to non-selective and constitutive autophagic activity, selective cargo recognition in mammalian autophagy relies on the interaction with ATG8/LC3 promoting their preferential engulfment. Selective autophagy receptors (SARs), such as the best known SQSTM1/p62 (sequestosome 1) (Bjørkøy et al., 2005), neighbor of *BRCA1* gene 1 (NBR1) (Lamark et al., 2009), calcium binding and coiled-coil domain 2 (CALCOCO2) (von Muhlinen et al., 2012), optineurin (OPTN) (Korac et al., 2013) BCL2/adenovirus E1B 19 kDa protein-interacting protein 3 (BNIP3) and its ligand NIX (or BNIP3L) (Zhang and Ney, 2009) have been described. SARs undergo post-translational (phosphorylation, ubiquitination, acetylation and oligomerization) and structural modifications to fulfill their role in autophagy, or upon executing their role, for their own degradation (Gubas and Dikic, 2021).

Once the specific cargo is selected and attached to the membrane, the autophagosomal membrane seals into a double-layered vesicle and ultimately fuses with the lysosome. Like multivesicular bodies formation, autophagosome closure requires membrane scission and could rely on ESCRT (endosomal sorting complexes required for transport). However, unclosed autophagosomes could be still fused with lysosomes and content degraded but at lower rates (Knorr et al., 2015; Takahashi et al., 2018).

The process of fusion with lysosomes or autophagolysosome formation requires several components such as ATG8 proteins, PIs, small GTPases, tethering adaptors, Soluble NSF attachment protein (SNAP) proteins, integral lysosomal proteins (LAMP-2) and Ras-related protein (RAB) proteins (RAB5 and RAB7). SNARE (N-ethylmaleimide–sensitive factor attachment protein receptor) proteins include STX17, SNAP29 and VAMP8 and the homotypic fusion and protein sorting (HOPS) complex is also involved in the process. Docking and fusion is controlled by RAB5, RAB7 and LAMP-2 proteins (Tanaka et al., 2000; Bento et al., 2013; Lorincz and Juhasz, 2020). The presence of ATG8 proteins and PI at the autophagosomal membrane allows the recruitment of guanine exchanging factor (GEF) Mon1-Ccz1 (Monensin sensitivity protein 1-Caffeine, calcium, and zinc 1), which also promotes association of the small GTPase Rab7 (Hegedűs et al., 2016). Both Rab7 and Rab2A, among others, seem to be indispensable for fusion with lysosomes in mammals (Gutierrez et al., 2004; Lorincz et al., 2017). Connections between the autophagosome and the lysosomal membrane are mediated by the tethering complex HOPS, which is able to catch Rab7-positive autophagosomes through binding with adaptor proteins. Finally, HOPS allows the connections with the SNARE complex, which mediates fusion of membranes (Lorincz and Juhasz, 2020). In particular, SNARE protein STX17 locates at the autophagosome membrane and constitutes a requirement for fusion with lysosomes (Itakura et al., 2012). The release of ATG8 proteins from the autophagosome membrane is mandatory for autophagolysosome formation (Yu et al., 2012). After fusion, the newly formed autophagolysosome uses acidic hydrolases to degrade autophagic cargos, and nutrients are then returned to the cytoplasm for recycling.

## Non-Coding RNA

For decades, the major cellular function of RNA was considered to be an intermediate molecule in the transfer of genetic information from DNA to proteins. However, nowadays, it is known that the human genome encodes tens of thousands of ncRNA transcripts that do not code for proteins. ncRNAs can be classified into two groups based on their size. One group includes short RNAs less than 200 nucleotides (nt) in length, such as miRNAs that are small RNA (sRNA) molecules around 21–25 nt in length, as well as other classes such as piwi-interacting RNAs (piRNAs) (Bushati and Cohen, 2007). The other group includes lncRNAs of around 200 nt or more (Esteller, 2011). Several decades ago, it was discovered that miRNAs play a relevant role in gene silencing, among other functions (Fire et al., 1998). In addition to small RNAs, recent genome-wide surveys have revealed that many regions encode lncRNAs that play essential regulatory roles at transcriptional level, alternative splicing, transport, messenger RNA (mRNA) stability and translation, occasionally, by affecting miRNA expression (Derrien et al., 2012).

The transcription of intergenic miRNAs is usually regulated by their individual promoters. Differently, the transcription of intronic miRNAs relies on the expression of their host mRNAs. Mammalian miRNAs tend to cluster along the genome, which guarantees the coordinate expression of different miRNAs (Megraw et al., 2007; Xu and Wong, 2008). miRNAs are mainly transcribed by RNA polymerase II, which produces hundreds or thousands of nucleotide-long products called primary miRNAs (pri-miRNAs) (Lee et al., 2004). However, transcription of some miRNA types may depend on RNA polymerase III (pol III) (Borchert et al., 2006). In the nucleus, the DROSHA nuclease complex and its regulatory subunit DGCR8 cleaves pri-miRNAs into 60-70-nucleotide hairpins, known as precursor-miRNAs (pre-miRNAs). The metazoan pre-miRNAs can also be produced from spliced introns. After cleavage they can be recognized by XPO5 (exportin-5) and transported from nucleus to the cytoplasm. In the cytoplasm, RNase III DICER1 protein further cleaves the hairpin structure of pre-miRNAs, which leads to the formation of ∼21–25 nt long miRNA duplexes. Then. these duplexes load onto a complex called RNA-induced silencing complex (RISC). Argonaute (AGO) proteins are important components of the RISC complex which guide single-stranded mature miRNAs to their target mRNAs (Ha and Kim, 2014).

Silencing is a result of a blockage of translation, mRNA sequestration in P-bodies or mRNA degradation. The degree of complementarity between miRNA and mRNA target sequences through the miRNA response elements (MREs) is critical for the miRNA action. The presence of a perfect base pairing between the seed sequence (6–8 bases in the miRNA that orchestrate binding) of mature miRNAs and target sequences on mRNAs leads to cleavage of mRNAs by RISC-associated RNases, i.e., AGO proteins. In the case of partial complementarity, protein production from target mRNAs could be inhibited through blockage of the translation machinery on ribosomes. Alternatively, miRNAs may lead to the sequestration of target mRNAs in P-bodies, resulting in their deadenylation, decapping, and subsequent exonucleolytic digestion. miRNAs usually regulate the expression of several different transcripts at once (Schwarz et al., 2003).

lncRNAs enclose a large and highly heterogeneous collection of transcripts that differ in their biogenesis and genomic origin. Statistics from Human GENCODE suggest that the human genome contains more than 16,000 lncRNA genes, but other estimates exceed 100,000 human lncRNAs (Uszczynska-Ratajczak et al., 2018). Most lncRNA species are transcribed by Pol II, and then many are often capped by 7-methyl guanosine at their 5′ ends, polyadenylated at their 3′ ends and spliced similarly to mRNAs. LncRNA genes are less evolutionarily conserved, contain fewer exons and are less abundantly expressed (Guo et al., 2020). Unlike mRNAs, many RNA polymerase II (Pol II)-transcribed lncRNAs are inefficiently processed, and are retained in the nucleus, whereas others are spliced and exported to the cytoplasm (Zuckerman and Ulitsky, 2019). The phosphorylation status of the Pol II carboxy-terminal domain corresponds with different transcription stages, and a significant fraction of lncRNAs are transcribed by phosphorylation-dysregulated Pol II, suggesting some of their other distinctive features (Schlackow et al., 2017). In addition to these general features of lncRNA transcription and processing mechanisms, lncRNAs often contain embedded sequence motifs that can recruit certain nuclear factors, which promote the nuclear localization and function of the lncRNA (Statello et al., 2021).

## Key Components in the Dual Role of Autophagy in Cancers

Autophagy plays a dual role in cancer initiation and progression depending on the cancer type, stage, and genetic context (Eisenberg-Lerner and Kimchi, 2009). Basal autophagy exerts a cytoprotective effect due to its ability to remove misfolded proteins and organelles and reactive oxygen species (ROS), thus contributing to avoid genomic damage which could lead to carcinogenesis. In this sense, autophagy is usually regarded as a suppressor mechanism during tumor initiation (White, 2012). Once tumors have been established, autophagy serves as a pro-tumoral mechanism that helps tumor cells to adapt to a stressful environment characterized by hypoxia, microenvironmental stimuli or nutrient starvation. Thus, autophagy allows to meet high metabolic requirements for tumor survival and faster proliferation, metastasis and therapeutic resistance (Yang et al., 2011; White, 2012; Qureshi-Baig et al., 2020). The interconnections between autophagy and apoptosis point out pro-apoptotic autophagy signaling as a desirable target for treatment design in cancer (Oral et al., 2016; Kocaturk et al., 2019).

*BECN1* encodes Beclin-1, which is a 60-kDa protein of 450 amino acids. It consists of three regulatory domains: the Bcl-2 homology 3 (BH3) domain at the N-terminus, a central coiled-coil domain (CCD) and a C-terminal evolutionarily conserved domain (ECD). It is frequently mono-allelically deleted in many human cancers such as breast, prostate, and ovarian cancers (Aita et al., 1999). Beclin-1 is an essential initiator of autophagy, and a key determining factor as to whether cells undergo autophagy or apoptosis. Bcl-2, Bcl-xL and Mcl-1 are well-known anti-apoptotic members of the Bcl-2 family that antagonize Bax and Bak, and thus prevent mitochondrial-dependent apoptosis. The regulation of Bcl-2/Bcl-xL interactions with Beclin-1 represents a central mechanism by which autophagy is regulated in response to diverse cellular stimuli (Levine et al., 2008) (Figure 1). This interaction prevents Beclin-1 from assembling the pre-autophagosomal structure, thereby inhibiting autophagy. HT-29 cells stably expressing Bcl-2 could inhibit starvation-induced autophagy via disrupting the Beclin-1/Vps34 complex (Pattingre et al., 2005). The inhibition of autophagy results from the Bcl-2:Beclin-1 interaction in the ER, as well as Bcl-2 sequestering autophagic components, such as AMBRA1, in mitochondria (Strappazzon et al., 2011) Different proteins such as, High mobility group box 1 (HMGB1) and BNIP3, compete for Bcl-2 and release Beclin-1, inducing autophagy (Bellot et al., 2009; Tang et al., 2010). The expression levels of Beclin-1 and Bcl-2 are key determinants of cell resistance to apoptosis or autophagy during carcinogenesis. Beclin-1 expression was lower in cancer cells (Kondo et al., 2005; Shi et al., 2009), heterozygous disruption of *BECN1* promotes tumorigenesis (Qu et al., 2003), and its overexpression inhibits tumorigenesis (Liang et al., 1999). *BECN1* is lost in 40–75% of breast and ovarian cancers (Liang et al., 1999). In fact, we have shown that the induction of apoptosis by a tyrosine kinase inhibitor was associated with an early full stimulation of ER-induced autophagy and c-Jun N-terminal protein kinase 1 (JNK) activation in liver cancer cells (Rodríguez-Hernández et al., 2018). The dissociation of Bcl-2:Beclin-1 complex can occur by phosphorylation of Bcl-2 by stress activated JNK1 (Wei et al., 2008). Death-associated protein kinase (DAPK) also phosphorylates a threonine within the BH3 domain of Beclin-1, inducing its dissociation from Bcl-2 and promoting autophagy (Zalckvar et al., 2009). Caspase-8-associated cleavage of Beclin-1 would ultimately disrupt the Bcl-2:Beclin1 complex, inactivate Beclin-1 and allow cells to undergo apoptosis (Li et al., 2011). It has been suggested that reduced expression of Beclin-1 reduces autophagic removal of damaged organelles that generate ROS and genotoxic stress, resulting in cellular transformation (Marquez and Xu, 2012).

Beclin-1 also regulates membrane trafficking events through its interaction with Vps15 and Vps34 with either ATG14L/BARKOR (ATG14; Complex I) or UVRAG (UV Radiation Resistance Associated, Vps38; Complex II) to regulate distinct vesicular trafficking functions (Kihara et al., 2001) (Figure 1). Complex I regulates autophagy, whereas Complex II regulates autophagy-independent functions including vacuolar protein sorting, cytokinesis, phagocytosis, fluid-phase endocytosis, or endolysosomal receptor trafficking (Galluzzi and Green, 2019; Liang et al., 2006). In particular, Beclin-1 may regulate tumor growth and progression through the control of endolysosomal trafficking of cell surface growth receptor function. Ligand binding initiates receptor internalization, entry in the early endosome compartment which is required for the activation of some signaling pathways (Cao et al., 2021), being later sorted to either late endosomes/multivesicular endosomes (MVEs) and sequestered within intraluminal vesicles (ILVs) for its degradation upon fusion with the lysosome (Dobrowolski and De Robertis, 2011), or to the recycling endosomes for return to the cell surface (Grant and Donaldson, 2009). Beclin-1, UVRAG, and Bax-interacting factor 1 (BIF-1) have been reported to regulate the rate at which the epidermal growth factor receptor (EGFR) is degraded after stimulation with its ligand epidermal growth factor (EGF) (Thoresen et al., 2010; Runkle et al., 2012). Beclin-1 expression inversely correlates with Akt and extracellular regulated kinase 1/2 (ERK) phosphorylation in human breast tumors through its Beclin-1-dependent regulation of insulin-like growth factor-1 (IGF-1) and EGF receptor trafficking (Rohatgi et al., 2015) (Figure 1). Beclin-1 controls endocytic receptor trafficking and tumor growth of EGFR and iron transporter transferrin receptor type 1 (TFR1) through coupling phosphatidylinositol 3-kinase catalytic subunit type 3 (PIK3C3), PI3P production, and phosphorylation and recruitment of hepatocyte growth factor tyrosine kinase substrate (HRS) to endosomes, mediating receptor sorting to the lysosome for degradation (Matthew-Onabanjo et al., 2020).

Similar to what has been observed with other ERBB family members, HER2 has also been reported to interact with Beclin-1 in breast cancer cells (Figure 1). Tyrosine kinase inhibitor lapatinib diminishes this interaction and induces autophagy (Han et al., 2013). The administration of a genetically engineered mutated isoform of Beclin-1 or Tat-Beclin-1 also protects from HER2-driven mammary tumorigenesis, and HER2 fails to inhibit autophagy in primary cells derived from these mice (Vega-Rubín-de-Celis et al., 2018). Interestingly, the administration of lapatinib on HER2, as well as erlotinib and nilotinib on EGFR and HER2 mutants, disrupts the interaction and phosphorylation between receptor and Beclin-1 and promotes autophagy (Vega-Rubín-de-Celis et al., 2020). Ligand-dependent fibroblast growth factor receptor 1 (FGFR1) activation also regulates total Beclin-1 levels and inhibits autophagy through the ERK-mitogen activated protein kinase (MAPK) pathway (Yuan et al., 2017).

### ER and Mitochondria-Dependent Structures Involved in Autophagosome Generation

The ER is the primary site for protein and lipid synthesis, membrane biogenesis, xenobiotic detoxification, and cellular Ca^2+^ storage. Cells exhibit a signaling system to restore homeostasis and normal ER function that include the unfolded protein response (UPR), ER-associated degradation (ERAD), autophagy, hypoxic signaling and mitochondrial biogenesis. Among the three ER-related branches, the activation of the protein kinase R (PKR)-like ER kinase (PERK) involves phosphorylation of eukaryotic translation initiation factor 2A (eIF2α) that reduce protein synthesis and induces upregulation of the Activating Transcription Factor 4 (ATF4) pathway that regulates the expression of a set of autophagy genes such as ATG16L1, ATG12, ATG3, Beclin-1, p62, ATG7, ATG10, ATG5, etc. (Senft and Ronai, 2015) Autophagy induced by ER stress mainly includes the ER stress-mediated autophagy and ER-phagy. The ER stress-mediated autophagy is characterized by the generation of autophagosomes that include worn-out proteins, protein aggregates, and damaged organelles, while the autophagosomes of ER-phagy selectively include ER membranes, and the double membranes also derive, at least in part, from the ER (Song et al., 2018).

Autophagosome formation occurs near ER subdomains enriched with phospholipid synthesizing enzymes like phosphatidylinositol synthase (Vega-Rubín-de-Celis et al., 2020)/CDP-diacylglycerol-inositol 3-phosphatidyltransferase (CDIPT) and choline/ethanolamine phosphotransferase 1 (CEPT1). ATG2, which transfers lipid molecules from the ER to form autophagosomes, vacuole membrane protein 1 (VMP1) and transmembrane protein 41b (TMEM41b) are ER membrane proteins that are associated with the formation of this subdomain (Yamamoto and Noda, 2020).

The intracellular site where autophagosome formation takes place is near the ER subdomain called the omegasome. Double FYVE-containing protein 1 (DFCP1) is a PI3P binding protein that serves as a specific marker of omegasome (Axe et al., 2008). Ultrastructural analyses have revealed that autophagosomes emerge from the vicinity of the ER (Hayashi-Nishino et al., 2009). Precisely, the ULK1 complex, a scaffold of ATG proteins, is localized in the tubular vesicular ER subdomain where autophagosome formation occurs (Karanasios et al., 2016).

The preservation of mitochondrial and ER functions is essential for the maintenance of cellular homeostasis. The ER makes physical contact with mitochondria at specific sites, and the hubs between the two organelles are called mitochondrial-associated ER membranes (MAMs). MAMs play key roles in biological processes, such as intracellular Ca^2+^ regulation, lipid trafficking and metabolism, as well as cell death, etc (Sun and Ding, 2020). Recent studies have suggested that MAMs are the key platform for autophagosome formation. As the marker of autophagosome formation, the majority of ATG5 is observed at the ER–mitochondria contact site during the entire autophagosome formation process. Autophagic process is also affected by the alteration of the MAM structure with the decrease of ATG14 and LC3-II (Hamasaki et al., 2013).

Ca^2+^ seems to be a relevant component in the ER-mitochondrial transducing signaling. Both the mitochondria and the ER have effective Ca^2+^ transport mechanisms to control both the internal and the external concentrations of the cation. Ca^2+^ controls many aspects of the physiological function of each organelle and is a major component of the apoptotic repertoire of both the mitochondria and the ER. Disruption of Ca^2+^ homeostasis is closely related to several pathologies and chronic diseases, such as cardiac diseases, Alzheimer’s disease, diabetes, etc (Walter and Hajnóczky, 2005). The majority of Ca^2+^ transportation between mitochondria and ER is performed through MAMs. Mitochondrial uptake of Ca^2+^ activates key enzymes of the Krebs cycle, thereby regulating mitochondrial ATP production. Therefore, MAMs effectively integrate Ca^2+^ flux with cellular metabolic pathways. However, excessive Ca^2+^ entrance into mitochondria induces the opening of mitochondrial permeability transition pore (mPTP) and release of pro-apoptotic factors, causing detrimental effects on cell viability (Rizzuto et al., 2012). MAM-mediated Ca^2+^ transfer from the ER to mitochondria may have either pro-survival or pro-death effects, perhaps contributing to enhanced mitochondrial ATP production to satisfy increased energy demands resulting from stress. Conversely, elevating mitochondrial Ca^2+^ may promote apoptosis (van Vliet et al., 2014).

## Role of lncRNA in Autophagy During Carcinogenesis

Abnormal expression of lncRNAs has been reported in numerous cancer types such as hematopoietic, urologic, lung, liver, breast, ovarian, or colorectal cancer (Sun et al., 2014; Shi et al., 2015; Xue et al., 2015; Zeng et al., 2015; Parasramka et al., 2016; Ricciuti et al., 2016). LncRNAs greatly impact autophagy at different carcinogenic stages, mostly in advanced metastatic stages. Most of lncRNAs positively affect autophagy ATGs proteins and their signaling pathways (Islam Khan et al., 2019). LncRNAs have been shown to eventually act as competing endogenous RNAs (ceRNAs) to modulate autophagy related miRNAs which can hijack miRNAs from target mRNAs (Islam Khan et al., 2019) (Table 1). Differently, other lncRNAs have an inverse relationship with autophagy, affecting different cell signaling impacting autophagy (Islam Khan et al., 2019) (Table 1). LncRNAs can modify autophagy from initiation to maturation. They can modulate autophagy phagophore initiation by upregulating mTOR, ULK1, ATG14L, Beclin-1, and autophagy phagophore elongation by upregulating ATG12, ATG5, ATG7, ATG4, and ATG3 (Zhang and Lu, 2020).

**TABLE 1:** Role of lncRNA in autophagy. Hepatocellular carcinoma, HCC; Gastric cancer, GC; Prostate cancer, PCa.

lncRNA	Type of cancer	Autophagy function	Mechanism	Reference
CASC9	Oral squamous cell carcinoma	Suppressed	Activation of Akt/mTOR pathway	Yang et al. (2019)
DCST1-AS1	HCC	Suppressed	Akt/mTOR signaling pathway modification	Li et al. (2019)
HAGLROS	GC	Suppressed	mTOR signal inhibition	Chen et al. (2018)
HNF1A antisense RNA 1	HCC	Promoted	miR-30b sponge, inhibits Bcl-2 expression and enhances ATG5 expression	Liu et al. (2016a)
HOTAIR	Colorectal cancer	Promoted	ATG12 upregulation by miR-93 sponging	Liu et al. (2020a)
HOTAIR	HCC	Promoted	Enhances ATG3, ATG7 expression	Yang et al. (2016)
HOTAIR	Cholangiocarcinoma	Suppressed	miR-204-5p sponge	Lu et al. (2020)
Nuclear Enriched Abundant Transcript 1 (NEAT1)	HCC	Promoted	Upregulates ATG3 expression by sponging miR-204	Li et al. (2020a)
SNHG1	PCa	Promoted	EZH2 targeting and activation of Wnt/β-catenin and PI3K/AKT/mTOR signaling pathways	Chen et al. (2020a)
ZNNT1	Uveal melanoma	Promoted	Upregulation of ATG12	Li et al. (2020b)

The HOX transcript antisense intergenic RNA (HOTAIR) has been associated with reduced expression of several miRNAs in different oncogenic settings. HOTAIR/miR-326/FUT6 axis modifies α1, 3-fucosylation of CD44, which triggers phosphatidylinositol 3 kinase (PI3K)/Akt/mTOR pathway mediating colorectal cancer progression (Pan et al., 2019a). HOTAIR knockdown and miR-203a-3p upregulation in colorectal cancer cell lines leads to lowered Wnt/β-catenin signaling, cell proliferation, and reduced chemoresistance (Xiao et al., 2018). Regarding autophagy, HOTAIR has shown different functions according to the cancer context. In cholangiocarcinoma, HOTAIR modulates miR-204-5p/HMGB1 axis supporting proliferation and reducing cell death and autophagy (Lu et al., 2020). On the other hand, HOTAIR depletion has also been demonstrated to inhibit cell autophagy in colorectal cancer due to its miR-93 sponging capacity, which regulates ATG12 expression. Therefore, ATG12 expression is upregulated upon miR-19 sponged by HOTAIR, increasing autophagy (Liu et al., 2020a). LncRNA HOTAIR has been demonstrated to be overexpressed in hepatocellular carcinoma (HCC), also promoting autophagy in these cells by upregulating ATG3 and ATG7 at mRNA and protein levels (Yang et al., 2016). LncRNA nuclear enriched abundant transcript 1 (NEAT1) has been described in diverse cancers progression. In HCC, NEAT1 upregulation promotes autophagy. It interacts with miR-204/ATG3 axis, sponging miR-204 and promoting ATG3 expression (Li et al., 2020a). Furthermore, in HCC HNF1A-AS1 functions as an oncogene and autophagy promoter through sponging miR-30b-5p, increasing Bcl-2 and ATG5 expression (Liu et al., 2016a).

Another lncRNA called ZNF706 Neighboring Transcript 1 (ZNNT1) plays role as potential tumor suppressor in uveal melanoma. ZNNT1 induces autophagy by upregulating ATG1*2* expression, and its downregulation causes an attenuation of an ATP-competitive kinase inhibitor of mTORC1 and mTORC2 mediated-autophagy (Li et al., 2020b).

LncRNA Small Nucleolar RNA Host Gene 1 (SNHG1) activates Wnt/β-catenin and PI3K/Akt/mTOR signaling pathways by targeting EZH2 (Enhancer of zeste homolog 2), and promoting proliferation, apoptosis and autophagy of prostate cancer (PCa) cells (Chen et al., 2020a). Other lncRNA that is upregulated in different cancers such as oral squamous cell carcinoma is cancer susceptibility candidate 9 (CASC9). A recent study showed that downregulation of CASC9 reduces Akt and mTOR phosphorylation, thereby promoting autophagy. Hence, they concluded that CASC9 acts as an oncogene by suppressing autophagy through the activation of the Akt/mTOR pathway (Yang et al., 2019). LncRNA DC-STAMP domain containing 1-antisense 1 (DCST1-AS1) is overexpressed in HCC. A study has demonstrated how DCST1-AS1 depletion promotes autophagy by its interaction with Akt/mTOR signaling pathway. Consequently, this lncRNAs inhibits autophagy by promoting Akt/mTOR signaling (Li et al., 2019). In gastric cancer (GC), signal transducer and activator of transcription 3 acute-phase response factor (STAT3) induced lncRNA HAGLROS overexpression inhibits autophagy by mTOR signal-mediated inhibition, thus contributing to the malignant proliferation and invasion of GC cells and consequently predicting poorer outcomes in GC patients (Chen et al., 2018).

## Role of miRNA in Autophagy During Carcinogenesis

### Regulation of Akt-mTOR Components by miRNA

**FIGURE 2:**
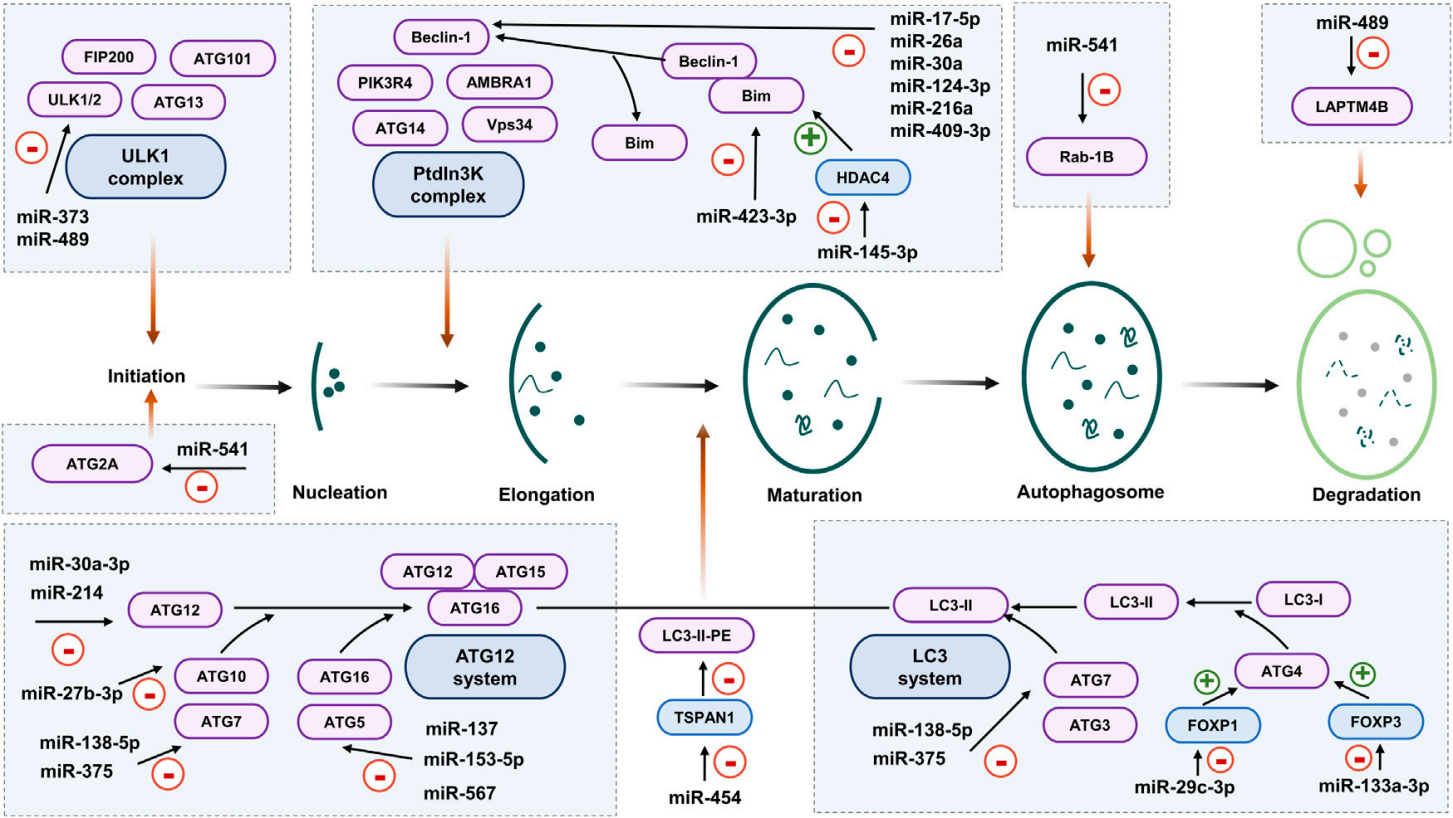
miRNA regulation of autophagic components. During autophagy initiation, the Unc-51 like autophagy activating kinase (ULK) complex activity could be diminished by miR-489 or miR-373, which target ULK1. Also, ATG2A could be negatively regulated by miR-541. Beclin-1 is targeted by miR-17-5p, miR-26a, miR-30a, miR-124-3p, miR-216a, and miR-409-3p. Beclin-1 activity is controlled by its interaction with Bim. In this sense, miR-423-3p increases autophagy activity by targeting Bim and miR-145-5p activates autophagy by downregulating histone deacetylase 4 (HDAC4), which controls Bim expression. The ATG12 conjugation system is tightly regulated by miRNAs. ATG12 is negatively regulated by miR-30a-3p and miR-214; ATG10 is targeted by miR-27b-3p; ATG7 is controlled by miR-138-5p and miR-375; and ATG5 expression is regulated by miR-137, miR-153-5p and miR-567. Lipidation of LC3 is potentiated by miR-454, that targets tetraspanin 1 (TSPAN1) and increases its turnover. ATG4 expression is negatively regulated by miR-29c-3p and miR-133a-3p through Forkhead box gene P1 (FOXP1) and Forkhead box gene P3 (FOXP3), respectively. Later stages of autophagy are less regulated. miR-541 participates in autophagosome competition by means of Rab-1B. miR-489 participates in degradation through targeting lysosomal protein transmembrane 4 beta (LAPTM4B).

The stimulation of Akt and mTOR signaling blocks autophagy. Different components of this survival pathway are targeted by miRNAs leading to upregulation of autophagy (Table 2; Figure 2). In particular, miR-338 mainly functions as a tumor suppressor in different cancers, in which its expression is reduced leading to an upregulation of Wnt-β-catenin, MAPK, and PI3K/Akt signaling pathways (Lu et al., 2018). Further mechanistic studies have identified miR-338 targets that activate the activating transcription factor 2 (ATF2), a histone acetyltransferase (HAT) that specifically acetylates histones H2B and H4 and activates transcription. The administration of an inhibitor of miR-338 reduces p-mTOR and p-p70S6 (phospho p70 S6 Kinase), leading to upregulation of autophagy, cell proliferation, colony formation and migration capabilities in cervical cancer models (Lu et al., 2018). Hypoxia inducible factor (HIF-1α) expression leads to reduced levels of miR-338-3p, which are responsible for hypoxia‐induced cell growth, migration, and epithelial-to-mesenchymal transition (EMT) in breast cancer (He et al., 2020). miR-139 mediates Akt/mTOR pathway inhibition and enhances autophagy in prostate cancer cells (Nam et al., 2019; Nam et al., 2020). Transient transfection with miR-139 mimics reduces mTOR expression at mRNA and protein levels, which leads to increased LC3-II/I conversion and Beclin-1 phosphorylation, but leads to autophagy blockade as seen by p62 increase, which could be responsible for increased apoptosis and reduced proliferation (Nam et al., 2020). The effect of miR-139 on apoptosis was confirmed by the cleavage of poly (ADP-ribose) polymerase 1 (PARP-1) and activation of p38 MAPK and ERK1/2 signaling (Nam et al., 2019; Nam et al., 2020). Therefore, upregulation of miR-139 could be seen as a potential therapeutic target in prostate cancer. The expression of miR-381 also appears to be downregulated in prostate cancer (Liao and Zhang, 2020). In this setting, the increased expression of its target reelin (RELN), an upstream regulator of the PI3K/Akt/mTOR axis, reduces the expression of Beclin-1, reduces LC3-II/I conversion and accumulates p62 (Liao and Zhang, 2020).

**TABLE 2:** Role of miRNA in autophagy. Hepatocellular carcinoma, HCC; Gastric cancer, GC; Prostate cancer, PCa.

miRNA	Type of cancer	Expression	Target	Role in autophagy	Mechanism	References
miR-7	Pancreatic cancer	Upregulated-Anti-tumoral	LKB1, ULK2, ATG4, ATG7	Upregulation suppresses autophagy	Modulation of AMPK/mTOR pathway	Gu et al. (2017)
					Degradation of ULK2, ATG4 and ATG7 mRNA degradation	
miR-7-5p	Cervical cancer	Upregulated-Pro-tumoral	Bcl-2	Upregulation promotes autophagy	Bcl-2 regulation	Yang et al. (2018)
miR-17-5p	Glioma	Downregulated-Pro-tumoral	Beclin-1	Downregulation promotes autophagy	Beclin-1 mRNA degradation	Hou et al. (2017)
miR-17-5p	Thyroid cancer	Upregulated-Pro-tumoral	PTEN	Upregulation promotes autophagy	Modulation of Akt-mTOR pathway	Shi et al. (2020)
miR-21	GC	Upregulated-Pro-tumoral	Not explored	Upregulation suppresses autophagy	Modulation of PI3K/Akt/mTOR pathway	Gu et al. (2020)
miR-21	Cervical cancer	Upregulated-Pro-tumoral	LATS1	Upregulation suppresses autophagy	Modulation of PI3K/Akt/mTOR pathway	Liu et al. (2015), Song et al. (2016)
miR-21	HCC	Upregulated-Pro-tumoral	PTEN	Upregulation suppresses autophagy	Modulation of PI3K/Akt/mTOR pathway	He et al. (2015)
miR-21	Breast cancer	Upregulated-Pro-tumoral	PTEN	Upregulation suppresses autophagy	Modulation of PI3K/Akt/mTOR pathway	Yu et al. (2016)
miR-21	Glioblastoma	Upregulated-Pro-tumoral	Not explored	Upregulation suppresses autophagy	Modulation of PI3K/Akt/mTOR pathway	Gwak et al. (2012)
miR-22	Osteosarcoma	Upregulated-Pro-tumoral	HMGB1	Upregulation suppresses autophagy	Modulation of PI3K/Akt/mTOR pathway	Meng et al. (2020), 187
miR-26a	Retinoblastoma	Downregulated-Pro-tumoral	Beclin-1	Downregulation promotes autophagy	Beclin-1 mRNA degradation	Li et al. (2016)
miR-27b-3p	Colorectal cancer	Downregulated-Pro-tumoral	ATG10	Downregulation promotes autophagy	ATG10 mRNA degradation	Sun et al. (2020)
miR-29c-3p	Ovarian cancer	Downregulated-Pro-tumoral	ATG14	Downregulation promotes autophagy	ATG14 mRNA degradation	Hu et al. (2020)
miR-30a	Osteosarcoma and gastrointestinal cancer	Downregulated-Pro-tumoral	Beclin-1	Downregulation promotes autophagy	Beclin-1 mRNA degradation	Xu et al. (2016), Chen et al. (2020b)
miR-30a-3p	Renal cell carcinoma	Downregulated-Pro-tumoral	ATG12	Downregulation promotes autophagy	ATG12 mRNA degradation	Chen et al. (2020c)
miR-34a	Breast cancer	Downregulated-Pro-tumoral	Bcl-2	Downregulation suppresses autophagy	Bcl-2 regulation	Li et al. (2013)
miR-34a	PCa	Downregulated-Pro-tumoral	ATG4B	Downregulation promotes autophagy	Modulation of AMPK/mTOR pathway	Liao et al. (2016)
miR-34a	Colorectal cancer	Downregulated-Pro-tumoral	IL6-R	Downregulation suppresses autophagy	Modulation of STAT3 signaling	Rokavec et al. (2014)
miR-106a	Cervical cancer	Upregulated-Pro-tumoral	LKB1	Upregulation suppresses autophagy	Modulation of AMPK/mTOR pathway	Cui et al. (2020)
miR-124-3p	Breast cancer	Downregulated-Pro-tumoral	Beclin-1	Downregulation promotes autophagy	Beclin-1 mRNA degradation	Zhang et al. (2016)
miR-133a-3p	GC	Upregulated-Pro-tumoral	FOXP3	Upregulation promotes autophagy	FOXO signaling	Li et al. (2020c)
miR-136-5p	Laryngeal squamous cell carcinoma	Upregulated-Anti-tumoral	ROCK1	Upregulation promotes autophagy	Modulation of PI3K/Akt/mTOR pathway	Yang et al. (2021)
miR-137	Pancreatic cancer	Downregulated-Pro-tumoral	ATG5	Downregulation promotes autophagy	ATG5 mRNA degradation	Wang et al. (2019)
miR-138	Lung cancer	Downregulated-Pro-tumoral	Sirt1	Downregulation promotes autophagy	Modulation of AMPK/mTOR pathway	Ye et al. (2017)
miR-138-5p	Lung cancer	Downregulated-Pro-tumoral	ATG7	Downregulation promotes autophagy	ATG7 mRNA degradation	Pan et al. (2019b)
miR-139	PCa	Upregulated-Anti-tumoral	IGF1R and AXL	Upregulation promotes autophagy	Modulation of mTOR pathway	Nam et al. (2019), Nam et al. (2020)
miR-140-3p	GC	Downregulated-Pro-tumoral	Bcl-2	Downregulation suppresses autophagy	Bcl-2 regulation	Chen et al. (2021)
miR-145-3p	Multiple myeloma	Downregulated-Pro-tumoral	HDAC4	Downregulation suppresses autophagy	HDAC4 mRNA degradation. Bim expression regulation	Wu et al. (2020)
miR-146b	Thyroid cancer	Upregulated-Pro-tumoral	PTEN	Upregulation promotes autophagy	Hyperactivation of PI3K/AKT signaling and Bcl-2 upregulation	Ramírez-Moya et al. (2018)
miR-153-3p	Lung cancer	Downregulated-Pro-tumoral	ATG5	Downregulation promotes autophagy	ATG5 mRNA degradation	Zhang et al. (2019)
miR-182	PCa	Upregulated-Anti-tumoral	Bcl-2	Upregulation promotes autophagy	Bcl-2 regulation	Peng et al. (2013)
miR-211	Cervical cancer	Downregulated-Pro-tumoral	Bcl-2	Downregulation suppresses autophagy	Bcl-2 regulation	Liu et al. (2020b)
miR-214	Colorectal cancer	Downregulated-Pro-tumoral	ATG12	Downregulation promotes autophagy	ATG12 mRNA degradation	Hu et al. (2018)
miR-216a	Pancreatic cancer	Downregulated-Pro-tumoral	Beclin-1	Downregulation promotes autophagy	Beclin-1 mRNA degradation	Zhang et al. (2015)
miR-338	Cervical cancer	Downregulated-Pro-tumoral	Not explored	Downregulation promotes autophagy	Inhibition of PI3K/Akt/mTOR axis	Lu et al. (2018)
miR-338-3p	Breast cancer	Downregulated-Pro-tumoral	ZEB2	Downregulation promotes autophagy	Modulation of Akt-mTOR pathway	He et al. (2020)
miR-346	Cervical cancer	Upregulated-Pro-tumoral	GSK3B	Upregulation promotes autophagy	Bcl-2 regulation	Guo et al. (2018)
miR-373	Cholangiocarcinoma	Downregulated-Pro-tumoral	ULK1	Downregulation promotes autophagy	ULK1 mRNA degradation	Lv et al. (2020)
miR-375	HCC	Downregulated-Pro-tumoral	ATG7	Downregulation promotes autophagy	ATG7 mRNA degradation	Chang et al. (2012)
miR-375	HCC	Downregulated-Pro-tumoral	ATG7	Downregulation promotes autophagy	Modulation of PI3K/Akt/mTOR pathway	Yuan et al. (2018)
miR-381	PCa	Downregulated-Pro-tumoral	RELN	Downregulation suppresses autophagy	Modulation of PI3K/Akt/mTOR pathway	Liao and Zhang, (2020)
miR-409-3p	Colorectal cancer	Downregulated-Pro-tumoral	Beclin-1	Downregulation promotes autophagy	Beclin-1 mRNA degradation	Tan et al. (2016)
miR-423-3p	GC	Upregulated-Pro-tumoral	Bim	Upregulation promotes autophagy	Bim mRNA degradation	Kong et al. (2017)
miR-451a	Breast cancer	Upregulated-Anti-tumoral	Not explored	Upregulation suppresses autophagy	Modulation of PI3K/Akt/mTOR pathway	Liu et al. (2016b)
miR-454	Pancreatic cancer	Downregulated-Pro-tumoral	TSPAN1 and FAM83A	Downregulation promotes autophagy	TSPAN1 and FAM83A mRNA degradation. Regulation of TSPAN1-LC3 interaction	Zhou et al. (2020)
miR-489	Breast cancer	Downregulated-Pro-tumoral	ULK1 and LAPTM4B	Downregulation promotes autophagy	ULK1 and LAPTM4B mRNA degradation	Soni et al. (2018)
miR-506	Pancreatic cancer	Upregulated-Anti-tumoral	STAT3	Upregulation promotes autophagy	STAT3-Bcl-2 regulation	Sun et al. (2017)
miR-519d	HCC	Downregulated-Pro-tumoral	Rab10	Downregulation suppresses autophagy	Modulation of AMPK/mTOR pathway	Zhang et al. (2020)
miR-519a	Glioblastoma	Downregulated-Pro-tumoral	STAT3^**^	Downregulation suppresses autophagy	STAT3-Bcl-2 regulation	Li et al. (2018)
miR-541	HCC	Downregulated-Pro-tumoral	ATG2A and RAB1B	Downregulation promotes autophagy	ATG2A and RAB1B mRNA degradation	Xu et al. (2020)
miR-547	Breast cancer	Downregulated-Pro-tumoral	ATG5	Downregulation promotes autophagy	ATG5 mRNA degradation	Han et al. (2020)
miR-1265	GC	Downregulated-Pro-tumoral	CAB39	Downregulation promotes autophagy	Modulation of AMPK/mTOR pathway	Xu et al. (2019)
miR-1910-3p	Breast cancer	Upregulated-Pro-tumoral	MTMR3	Upregulation promotes autophagy	Activation of the NF-κB signaling pathway and Bcl-2 upregulation	Wang et al. (2020)

**miRNA target not confirmed by luciferase assays.

The oncomir miR-17-5p targets anti-tumoral phosphatase and tensin homolog (PTEN), which controls activation of the Akt-mTOR pathway in thyroid cancer (Shi et al., 2020) (Table 2). The inhibition of miR-17-5p reduces proliferation and autophagy but increases cell death by apoptosis in thyroid cancer. miR-146b also targets PTEN, leading to hyperactivation of PI3K/Akt signaling and apoptosis protection by upregulation of anti-apoptotic Bcl-2 (Ramírez-Moya et al., 2018). The expression of miR-375 is also reduced in GC (Yuan et al., 2018). In fact, the overexpression of miR-375 leads to decreased proliferation, migration, invasion, and autophagy by reducing phosphorylation of Akt and mTOR. These results were confirmed in an *in vivo* model with nude mice, showing miR-375 suppression of tumor formation correlated with reduced Akt signaling (Yuan et al., 2018). In this model, miR-375 suppresses cytoprotective pathways in GC. However, concomitant autophagy reduction and Akt-mTOR phosphorylation suggests other regulators of autophagy.

### Regulation of AMPK by miRNA

AMPK is a major controller of lipid metabolism, regulating cholesterol and fatty acid levels (Habegger et al., 2012), and energy metabolism, activated by depletion of ATP, low glucose or changes of NADPH levels (Jeon et al., 2012) to maintain cellular energy homeostasis. AMPK is seen as a tumor suppressor, downregulating mTOR, upregulating p53 and promoting cell cycle arrest (Li et al., 2015). miRNAs have also been shown to upregulate AMPK signaling and oncogenic autophagy. Calcium-binding protein 39 (CAB39), MO25α, forms a heterotrimeric complex with a ste20-related adaptor and liver kinase B1 (LKB1), being this complex a major upstream regulator of AMPK in mammalian cells (Boudeau et al., 2003). miR-1265 suppresses GC progression and oncogenic autophagy by reducing CAB39 expression and regulating the AMPK-mTOR signaling pathway (Xu et al., 2019). The antitumoral profile of miR-1265 has been demonstrated in different *in vitro* and *in vivo* approaches, showing that its overexpression reduces tumor growth (Xu et al., 2019). The overexpression of miR-138 reduces mTOR phosphorylation, cell proliferation, EMT markers and autophagy, and induces cell cycle arrest in G1/S phase in non-small cell lung cancer (NSCLC) (A549 and Calu-3) (Ye et al., 2017). As miR-1265 and miR-138, miR-519d also exerts an antitumor effect in HCC through Rab10 and AMPK signaling pathway. In particular, HCC shows low levels of miR-519d, which correlate with higher expression of its target Rab10. Either overexpression of miR-519d or Rab10 silencing reduces proliferation and induces apoptosis and autophagy through AMPK. miRNA mimics triggers expression of Bak, Beclin-1, ATG5, and p53, LC3-II/I conversion and phosphorylated AMPK, while decreases Rab10, mTOR and Bcl-2 (Zhang et al., 2020). Otherwise, the oncomir miR-106a is significantly upregulated in human papiloma virus 16-induced cervical cancer, fostering proliferation and reducing autophagy (Cui et al., 2020). The pro-survival functions of miR-106a are mediated by targeting LKB1, thus preventing AMPK phosphorylation and increasing mTOR signaling (Cui et al., 2020). LKB1 is also regulated by miR-7 in pancreatic cancer in which it has been shown that miR-7 overexpression reduces AMPK activation and promotes mTOR signaling with consequent autophagy inactivation, and reduced Warburg effect in pancreatic cancer cells. This strategy displays lower migration and invasion rates (Gu et al., 2017) (Table 2).

### Impact of miRNA on Treatment Resistance

miRNAs could also involve treatment resistance through autophagy. For instance, miR-21 has been widely studied as an oncomir that promotes treatment resistance by downregulating autophagy. In GC, resistance to cisplatin-based chemotherapy seems to be controlled by overexpression of miR-21. In cisplatin resistant GC cells, miR-21 expression correlates with decreased autophagy and activation of the PI3K/Akt/mTOR axis (Gu et al., 2020). A similar mechanism of resistance has also been proved in cervical cancer by targeting large tumor suppressor kinase 1 (LATS1). In this context, miR-21 participates in a positive feedback loop which involves PTEN-Akt-HIF-1α (Liu et al., 2015; Song et al., 2016). Similarly, miR-21 is also overexpressed in Sorafenib resistant HCC cell lines. This leads to decreased autophagy by activation of pro-survival pathways that decrease PTEN expression (He et al., 2015). In fact, the increased tumor expression of miR-21-3p correlates with poor overall survival in patients with HCC. SMAD7 targeting by miR-21-3p is responsible for its pro-tumoral effects. In particular, miR-21-3p mimics promote cell migration, invasion and EMT (Hong et al., 2021). Therefore, silencing of miR-21 should be highlighted as a promising therapeutic approach. As a matter of fact, blockade of miR-21 in estrogen receptor-alpha positive (ER+) breast cancer lines with miRNA inhibitors leads to higher therapeutical benefits, as seen by induction of autophagic cell death through inhibition of the PI3K-Akt-mTOR pathway (Yu et al., 2016). Similarly, silencing of miR-21 in malignant glioma cell lines contributes to decreased autophagy and sensitizes to γ-irradiation (Gwak et al., 2012).

The downregulation of other miRNAs has also been related to therapy resistance. In fact, miR-451a reduces Akt and mTOR phosphorylation, leading to anti-proliferative and pro-apoptotic activity in breast cancer cells (Liu et al., 2016b). Other miRNAs participate in cisplatin treatment response by altering autophagy. For example, miR-22 regulates cisplatin resistance in osteosarcoma by reducing autophagy (Meng et al., 2020). On the other hand, miR-136-5p plays an anti-tumoral role targeting Rho Associated Coiled-Coil Containing Protein Kinase 1 (ROCK1) that attenuates Akt activation and promotes autophagy, while reducing cell migration and invasion in head and neck cancer cells (Yang et al., 2021). Chemosensitivity could be also regulated by the AMPK-mTOR axis. In this sense, the downregulation of miR-34a in prostate cancer involves chemoresistance through autophagy promotion by means of regulation of AMPK and different autophagy components such as 4B cysteine peptidase (ATG4B), which appears to be a direct target of miR-34a (Liao et al., 2016). In prostate cancer, miR-34a expression is downregulated by hypermethylation both in patient derived tissues and in cell lines. The study also shows that the *in vitro* upregulation of miR-34a leads to increased sensitivity to doxorubicin and topotecan and reduced autophagy in cell lines (Liao et al., 2016).

### Impact of miRNAs on Bcl-2 Expression and its Relation to Treatment Resistance

STAT3 signaling constitutes an important upstream regulator of Bcl-2 expression. Nuclear STAT3 negatively regulates autophagy mainly through upregulating anti-apoptotic genes such as Bcl-2 or Mcl-1 (Bhattacharya et al., 2005), and downregulating pro-autophagic Beclin-1 (Miao et al., 2014), but also PIK3C3, CTSB, CTSL, PIK3R1, HIF-1α, BNIP3, and microRNAs with targets of autophagy modulators (You et al., 2015). Cytoplasmic STAT3 constitutively inhibits autophagy by sequestering eukaryotic translation initiation factor 2-alpha kinase 2 (EIF2AK2) as well as by interacting with other autophagy-related signaling molecules such as Forkhead box O1 (FOXO1) and Forkhead box O3 (FOXO3) (You et al., 2015). Constitutive activation of STAT3 signaling is required for the maintenance of malignant tumors. miR-34a participates in a feedback loop in which interleukin-6 (IL-6) mediates the repression of miR-34a, leading to the activation of IL-6R and STAT3, which in turns maintains the repression of miR-34a that helps to support a mesenchymal pro-metastatic phenotype in colorectal cancer cells (Rokavec et al., 2014). Furthermore, the downregulation of miR-34a in breast cancer is related to elevated expression of Bcl-2 and sirtuin-1 (Sirt1), and consequently the restoration of miR-34a expression leads to increased apoptosis rates (Li et al., 2013). The downregulation of miR-519a has been related to upregulation of STAT3/Bcl-2 signaling and reduced autophagy in temozolomide resistant glioblastoma cells (Li et al., 2018). The resistance to treatment is overcome by miR-519a overexpression leading to increased expression of Beclin-1 and LC3B, as well as accumulation of LC3B after Bafilomycin A1 (BafA1) treatment in resistant cells (Li et al., 2018). In the same way, the ectopic expression of miR-506 suppresses STAT3 signaling and reduces Bcl-2 expression, while increases Beclin-1 expression and promotes autophagy-dependent cell death in pancreatic cancer cells (Sun et al., 2017).

Other miRNAs that directly regulate Bcl-2 expression to control apoptosis and autophagy in cancer cells are shown in Table 2. In this sense, the downregulation of miR-211 increases expression of its target Bcl-2 and reduces apoptotic and autophagic rates in cervical tumors (Liu et al., 2020b). In cisplatin resistant cervical cancer cells, the targeting of PARP-1 by miR-7-5p appears to be responsible for the reduction of apoptotic rates that is associated with upregulation of energy production and autophagy through Bcl-2 downregulation (Yang et al., 2018). miR-346 also plays a pro-survival role in cervical cancer upon activation of ER stress, reduction of Bcl-2 and release of Beclin-1, induction of autophagy and mitophagy and reduction of ROS and apoptosis (Guo et al., 2018). The upregulation of miR-182 by nutritional stress including atorvastatin treatment or serum withdrawal leads to downregulation of Bcl-2 and increases autophagy in prostate cancer cells (Peng et al., 2013). Similarly, miR-140-3p regulates cell migration, invasion and autophagic cell death by regulating Bcl-2 and Beclin-1 in GC (Chen et al., 2021). miR-1910-3p participates in breast cancer cells proliferation and metastasis through indirect autophagy regulation. MCF7 and MDA-MB-231 cells upregulate miR-1910-3p in response to lipopolysaccharide (LPS). The transient transfection with miR-1910-3p mimic or exosomal transference increases cell migration, colony formation capabilities and proliferation in breast cancer cells (Wang et al., 2020). Furthermore, miR-1910-3p promotes EMT transition through upregulation of N-cadherin and vimetin and downregulation of E-cadherin (Wang et al., 2020).

Bim is a proapoptotic BH3-only Bcl-2 family member that interacts with Beclin-1 and prevents autophagy (Luo et al., 2012). High circulating levels of miR-423-3p constitute a biomarker of poor outcome and associate with clinicopathological characteristics of GC (Kong et al., 2017). The reduction of Bim by miR-423-3p that promotes Beclin-1 release is associated with its pro-tumoral functions, involving activation of cell proliferation, autophagy, cell migration and invasion (Kong et al., 2017). miR-145-3p, which is downregulated in multiple myeloma, targets the histone deacetylase 4 (HDAC4) that regulates, among other genes, the transcription of *BIM*. In consequence, low miR-145-3p expression is associated with reduced Bim expression, upregulation of mTOR and cell survival (Wu et al., 2020).

### Regulation of Autophagic Components by miRNAs

Several miRNAs target the expression of autophagic components that impact in tumor progression and treatment resistance (Table 2 and Figure 2). miR-489 participates in sensitizing breast cancer cells to chemotherapy through ULK1 downregulation, as well as hampering autophagosome degradation by targeting the lysosomal protein transmembrane 4 beta (LAPTM4B) (Soni et al., 2018). The upregulation of miR-373 also targets ULK1 and reduces autophagy in cholangiocarcinoma (Lv et al., 2020).

Not only the initiation phase of autophagy could be regulated by miRNAs, but also the nucleation stage, in particular Beclin-1, which is one of the key players of autophagy in cancer. Several miRNAs have been shown to impact Beclin-1 functions and therefore, to impact on treatment response. In colorectal cancer, miR-409-3p downregulation has been associated with oxaliplatin resistance by increased autophagy mediated by its target Beclin-1 (Tan et al., 2016). In this context, autophagy exerts a pro-tumoral role favoring treatment resistance. Thus, overexpression of miR-409-3p might be considered as a good prognosis factor. Similarly, tumor progression in breast cancer is related to low expression of miR-124-3p that controls autophagy induction by means of Beclin-1 (Zhang et al., 2016). Other miRNAs, such as miR-216a, miR-17-5p, miR-26a or miR-30a, that specifically target Beclin-1, support the idea that induction of autophagy after cancer therapy triggers resistance (Zhu et al., 2009; Zhang et al., 2015; Li et al., 2016; Hou et al., 2017). One of the masters ncRNAs regulators of Beclin-1 is miR-30a, whose role has been described in several types of cancer and its overexpression has been regarded to have a positive impact on cancer progression (Xu et al., 2016; Chen et al., 2020b).

Treatment resistance could be related to autophagy initiation by affecting ATG14 pro-autophagic protein. Forkhead box gene P1 (FOXP1), a member of the FOXP subfamily of transcription factors, appears to be associated with malignancy (Koon et al., 2007). Cisplatin treatment resistance has been associated with downregulation of miR-29c-3p in ovarian cancer (Hu et al., 2020). Mechanistically, miR-29c-3p targets FOXP1 which controls the expression of ATG14. Downregulation of miR-29c-3p increases ATG14 expression and other autophagic markers such as Beclin-1 and LC3-II/LC3-I ratio, and reduces p62 expression in resistant cells (Hu et al., 2020). Similarly, miR-133a-3p targeting of 3′-UTR of Forkhead box gene P3 (FOXP3) increases cell proliferation and autophagy in GC (Li et al., 2020c).

ATG7 participates in the generation of ATG5-ATG12 complex and LC3 processing during autophagy elongation. miR-375 has a critical role during the induction of autophagy in liver cancer cells submitted to hypoxia. The overexpression of miR-375 reduces ATG7 expression, prevents mitophagy and contributes to cytochrome release and apoptosis in Huh7 and Hep3B (Chang et al., 2012). The downregulation of miR-541 leads to increased expression of ATG2A and Rab-1B, and autophagy in tumor cells correlates with a shorter recurrence free survival (RFS). miRNA mimics or transient inhibition of ATG2A or Rab-1B contributes to sensitizing HCC cells to Sorafenib treatment (Xu et al., 2020).

The tripartite motif-containing protein 65 (TRIM65) is an E3 ubiquitin ligase and a critical regulator of a variety of cellular processes as well as tumor progression (Chen et al., 2019). TRIM65 leads to reduced levels of miR-138-5p that targets ATG7 in lung tumor resistant cells (Pan et al., 2019b). Similarly, tetraspanin 1 (TSPAN1) is a direct regulator of autophagy by interacting with LC3, enhancing its turnover and the fusion of autophagosomes and lysosomes in an AMPK and mTOR independent manner. In turn, the expression of cancer-associated cell migration protein TSPAN1 expression is controlled by the Wnt-β-catenin 1 (CTNNB1) signaling pathway by means of the family with sequence similarity 83 member A (FAM83A). At the same time, TSPAN1 and FAM83A mRNA are controlled by miR-454, whose expression is reduced in pancreatic cancer. All in all, these results suggest that low levels of miR-454 contribute to pancreatic tumor malignancy by inducing autophagy flux (Zhou et al., 2020).

The ATG5-ATG12 complex is importantly regulated by miRNAs. The downregulation of miR-137 that targets ATG5 expression could be functioning as a doxorubicin resistance mechanism in pancreatic cancer (Wang et al., 2019). Trastuzumab resistance in breast cancer is also governed by increased ATG5 related autophagy. In particular, both non-responding patients and resistant cells display low expression levels of miR-567 that targets ATG5, and it has been shown that exosomal transfer of miR-567 might provide chemosensitivity and consequently to be considered as a potential therapeutic approach (Han et al., 2020). Equivalently, low expression of miR-153-3p in NSCLC correlates with gefitinib treatment resistance by upregulating autophagy mediated by ATG5 (Zhang et al., 2019). It has also been observed that after radiotherapy, miR-214 is downregulated, which upregulates ATG12 and autophagy. Therefore, miR-214 should be considered as a marker of radioresistance in colorectal cancer (Hu et al., 2018). Different *in vitro* studies and in clinical setting showed that higher expression of miR-30a-3p and lower ATG12 expression in renal cell carcinoma tissues correlates with reduced metastatic potential and better patient prognosis (Chen et al., 2020c). Another important factor regulating the ATG12-ATG5 complex formation and LC3 processing is ATG10. This autophagic protein is also modulated by miRNA expression during oxaliplatin resistance in colorectal cancer. In particular, low levels of miR-27b-3p are associated with higher expression of ATG10 and autophagy during oxaliplatin resistance in colorectal cancer (Sun et al., 2020).

## Concluding Remarks

Autophagy is a relevant cellular process involved in restoring homeostasis. In this sense, it can exert a dual function in cancer, being able to participate as a tumor suppressor mechanism at early stages but also as an oncogenic one at advanced stages of malignancy in order to readapt cell metabolism in cancer cells. In this setting, it should be considered as a major mechanism involved in treatment resistance in cancer cells. In a broad range of malignancies, the downregulation of miRNA expression has been shown to be related to upregulation of autophagic components and disease progression. Therefore, autophagy and upstream regulators including pro-survival signaling pathways and non-coding RNAs should be considered as targets to provide chemo or radiosensitivity in cancer therapies.

## Glossary

AMBRA-1: Activating molecule in Beclin1-regulated autophagy protein 1
ATF4: Activating Transcription Factor 4
AMPK: AMP-activated protein kinase
AGO: Argonaute
ATF2: Activating transcription factor 2
ATG: Autophagy related
ATG4B: 4B cysteine peptidase
BafA1: Bafilomycin A1
BH3: Bcl-2 homology 3
BIF-1: Bax-interacting factor 1
BNip3: BCL2/adenovirus E1B 19-kDa-interacting protein 3-like
Bnip3l: BNip3 ligand or NIX
CAB39: Calcium-binding protein 39
CALCOCO2: Calcium binding and coiled-coin domain 2
CASC9: Cancer susceptibility candidate 9
CMA: Chaperone-mediated autophagy
CTNNB1: β-catenin 1
CDIPT: CDP-diacylglycerol-inositol 3-phosphatidyltransferase
CEPT1: Choline/ethanolamine phosphotransferase 1
CCD: Coiled-coil domain
ceRNAs: Competing endogenous RNAs
DAPK: Death-Associated Protein Kinase
DCST1-AS1: DC-STAMP domain containing 1-antisense 1
DFCP1: Double FYVE domain-containing protein
EGF: Epidermal growth factor
ER: Endoplasmic reticulum
ESCRT: Endosomal sorting complexes required for transport
EZH2: Enhancer of zeste homolog 2
EGFR: Epidermal growth factor receptor
EMT: Epithelial-to-mesenchymal transition
ERAD: ER-associated degradation
ER+: Estrogen receptor-alpha positive
eIF2α: Eukaryotic translation initiation factor 2A
EIF2AK2: Eukaryotic translation initiation factor 2-alpha kinase 2
ECD: Evolutionary conserved domain
ERK 1/2: Extracellular regulated kinase 1/2
FAM83A: Family with sequence similarity 83 member A
FTH1: Ferritin heavy chain 1
FGFR1: Fibroblast growth factor receptor 1
FOXP1: Forkhead box gene P1
FOXP3: Forkhead box gene P3
FOXO1: Forkhead box O1
FOXO3: Forkhead box O3
GC: Gastric cancer
GATE-16: Golgi-associated ATPase Enhancer of 16 kDa
GABARAP: γ-aminobutyric acid receptor-associated proteins
GEF: Guanine exchanging factor
HSC70: Heat shock cognate 70 KDa protein
HCC: Hepatocellular carcinoma
HRS: Hepatocyte growth factor tyrosine kinase substrate
HAT: Histone acetyltransferase
HDAC4: Histone deacetylase 4
HIF: Hypoxia inducible factor
HOTAIR: HOX transcript antisense intergenic RNA, ;
HMGB1: High mobility group box 1
HOPS: Homotypic fusion and protein sorting
FIP200: Inducible coiled-coil protein 1
ILVs: Intraluminal vesicles
IGF-1: Insulin-like growth factor-1
IL-6: Interleukin-6
JNK: c-Jun N-terminal Kinase
LATS1: Large tumor suppressor kinase 1
LIR: LC3 interaction region
LPS: Lipopolysaccharide
LC3: Microtubule associated protein light chain 3
LKB: Liver kinase B
LncRNA: Long non-coding RNA
LAPTM4B: Lysosomal protein transmembrane 4 beta
LAMP-2: Integral lysosomal proteins
miRNA: MicroRNA
mTOR: Mammalian target of rapamycin
MTORC1: Mammalian target of rapamycin complex 1
mRNA: Messenger RNA
MREs: miRNA response elements
MAMs: Mitochondrial-associated ER membranes
mPTP: Mitochondrial permeability transition pore
MAPK: Mitogen activated protein kinase
Mon1-Ccz1: Monensin sensitivity protein 1-Caffeine, calcium, and zinc 1
MVEs: Multivesicular endosomes
NBR1: Neighbor of BRCA1 gene 1 protein
NSCLC: Non-small cell lung cancer
NEAT1: Nuclear enriched abundant transcript 1
PARP-1: Poly (ADP-ribose) polymerase 1
PCa: Prostate cancer
PC: Phosphatidylcholine
PERK: Protein kinase R (PKR)-like endoplasmic reticulum kinase
PE: Phosphatidylethanolamine
PKB: Protein kinase B
PI: Phosphoinositol lipids
PI3K: Phosphatidylinositol 3 kinase
PIK3C3: PI3K catalytic subunit type 3
PIP3: Phosphatidylinositol (3,4,5)-trisphosphate
PI3P: Phosphatidylinositol 3-phosphate
piRNA: Piwi RNA
pri-miRNA: Primary miRNA
pre-miRNA: Precursor miRNA
PS: Phosphatidylserine
PTEN: Phosphatase and tensin homolog
p-p70S6K: Phospho p70 S6 Kinase
pol II: RNA polymerase II
pol III: RNA polymerase III
RAB: Ras-related protein
RISC: RNA-induced silencing complex
ROS: Reactive oxygen species
RFS: Recurrence free survival
RELN: Reelin
ROCK1: Rho Associated Coiled-Coil Containing Protein Kinase
SNARE: N-ethylmaleimide–sensitive factor attachment protein receptor
SARs: Selective autophagy receptors
SNAP: Soluble NSF attachment protein
SNHG1: Small nucleolar RNA host gene 1
SQSTM1/p62: Sequestosome 1
STAT3: Signal transducer and activator of transcription 3 acute-phase response factor
Sirt1: Sirtuin-1
sRNA: Small RNA
STX17: Syntaxin17
TKR: Tyrosine kinase receptor
TSPAN1: Tetraspanin 1
TFR1: Iron transporter transferrin receptor type I
TMEM41b: Transmembrane protein 41b
TRIM65: Tripartite motif (TRIM)-containing protein 65
TSC1/2: Tuberous sclerosis complex 1 and 2
p53: Tumor protein p53
ULK: Unc-51 like autophagy activating kinase
UPR: Unfolded protein response
UVRAG: UV radiation resistance associated
Vps15: Vacuolar protein sorting 15
Vps34: Vacuolar protein sorting 34
VMP1: Vacuole membrane protein 1
WIPI2: WD repeat domain phosphoinositide-interacting protein
XPO5: Exportin-5
ZNNT1: ZNF706 Neighboring Transcript 1
